# Exclusion of Left Atrial Appendage Thrombus Using Single Phase Coronary Computed Tomography as Compared to Transesophageal Echocardiography in Patients Undergoing Pulmonary Vein Isolation

**DOI:** 10.1155/2014/838727

**Published:** 2014-02-20

**Authors:** Jason Saucedo, Shaun Martinho, Dianne Frankel, Ahmad M. Slim, Robert E. Eckart

**Affiliations:** Cardiology Service, Brooke Army Medical Center, 3551 Roger Brooke Drive, Fort Sam Houston, San Antonio, TX 78234-6200, USA

## Abstract

*Background*. Transesophageal echocardiography (TEE) is used for the evaluation of the presence of left atrial appendage (LAA) thrombus prior to pulmonary vein isolation (PVI), while coronary computed tomography angiography (CCTA) is used for anatomic mapping during PVI. *Methods*. We compared the diagnostic performance of single phase CCTA to TEE in excluding the presence of LAA thrombus in patients undergoing PVI in 172 subjects performed during index hospitalization. *Results*. The mean age was 51 ± 13 years, a median CHADS_2_ score of 1 [IQR_25,75_ 0,1, range 0–3] and a mean periprocedural INR of 2.1 ± 0.6. The prevalence of an LAA filling defect on single phase CCTA was 9.3% (6/183) and on TEE was 1.2% (2/183). Sensitivity, specificity, positive predictive value, and negative predictive value were 100% (95% CI, 19.8–100%), 91.8% (95% CI, 94–99%), 12.5% (95% CI, 60–76%), and 91.8% (95% CI, 97–100%) for the detection of LAA filling defect, respectively. *Conclusion*. Given the utility of a preprocedural single phase CCTA for the performance of PVI, the absence of a filling defect negates the need for a subsequent TEE as an adjunct for exclusion of LAA thrombus.

## 1. Background

Transesophageal echocardiography (TEE) historically is the gold standard for the evaluation of the presence of left atrial appendage thrombus in patients with atrial fibrillation. In a recent study, evaluating spontaneous echo contrast (SEC), left atrial (LA) appendage velocities, and aortic plaque TEE variables in the absence of definitive left atrial thrombus (LA) in five hundred and seventy-nine consecutive patients undergoing pulmonary vein isolation (PVI), all variables with the exception of LA thrombus were not predictive of the occurrence of stroke within thirty days on anticoagulation with Vitamin K Antagonist. [1]. This is in contrast to a smaller study of 156 consecutive patients, where functional parameters to include the emptying velocities and SEC were predictive of the presence of LAA thrombus on TEE [2].

Advances in coronary computed tomography angiography (CCTA) imaging have led to high spatial and temporal resolution. CCTA is emerging as a potential screening tool for exclusion of LAA thrombus [3]. Patients routinely undergo TEE before pulmonary vein isolation (PVI) to exclude thrombus and to assess pulmonary venous anatomy and single phase CCTA for incorporation into nonfluoroscopic anatomic mapping systems used during PVI [3].

A number of studies have assessed their application for determining the presence or absence of thrombus. Romero et al. completed a meta-analysis of detection of LAA thrombus by CCTA in patients with atrial fibrillation that demonstrated an overall high accuracy of CCT compared with TEE for the detection of LA/LAA thrombus. The mean sensitivity and specificity were 96% and 92% and positive predictive value and negative predictive value were 41% and 99% [4].

The purpose of this review is to evaluate the diagnostic accuracy of single phase CCTA obtained routinely in our institution prior to pulmonary vein isolation (PVI) in comparison to TEE in excluding the presence of LAA thrombus and thus limiting the need for invasive procedures such as TEE to only patients with possible thrombus on CCTA.

## 2. Methods

### 2.1. Study Selection

Chart review evaluating concomitant (within 48 hours) single phase 64- or 128-slice CCTA and TEE performed between January 2008 and June 2012 prior to PVI. All procedures were performed in symptomatic individuals with atrial fibrillation over the age of 18 at a single tertiary referral center (San Antonio Military Medical Center, Joint Base San Antonio-Fort Sam Houston, Texas). LAA was evaluated by both CCTA and TEE for filling defects. Imaging modalities were reviewed by a level II echocardiographer and a level III CT imaging staff to assess for the presence or absence of a thrombus.

**Figure 1:**
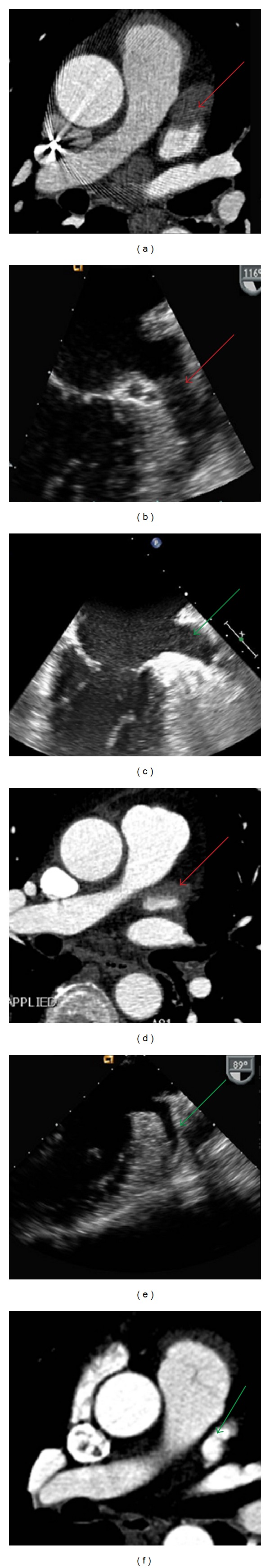
Comparison of CCTA to TEE. Red arrow = filling defect, green arrow = no filling defect. Images (a) and (b) show correlating filling defects as noted on CCTA and TEE in the same subject, while images (c) and (d) show discordance between the two modalities in a different subject, and finally images (e) and (f) demonstrate no defect on either modality as noted by correlating arrows.

From January of 2008 to March of 2011, images were obtained using a retrospective helical protocol with a 64-slice CT scanner (Somatom Definition CT, Siemens, Erlangen, Germany). From March of 2011 to March of 2012, studies were obtained utilizing a prospective sequential protocol with 60–80% image acquisition window. After March of 2012, 128-slice dual head scanner with a single heart beat image acquisition of the complete cardiac anatomy if a heart rate of less than 60 was achieved. (Somatom Definition Flash CT, Siemens, Erlangen, Germany). The subject's EE images were analyzed utilizing Merge Cardio (Merge Healthcare, Chicago, IL) for the evaluation of the entire left atrial appendage. The subject's CCTA images were evaluated using Vitrea 15 software (Vital Images, Inc., Minnetonka, MN) (Figure 1).

### 2.2. Statistical Analysis

Statistical analysis was performed using IBM SPSS version 19.0 (IBM, Armonk, NY). Continuous variables are presented as means ± standard deviation and medians with interquartile range, as appropriate. Categorical variables are presented as frequencies with percentages. Comparison of means was performed using one-way ANOVA with post hoc Bonferroni correction, with *P* values <0.05 considered significant.

## 3. Results

We reviewed 403 studies and excluded 231, 15 studies due to time intervals >48 hrs between imaging modalities, 60 studies were not included as neither a TEE nor CCTA was done concomitantly. We compared the diagnostic performance of single phase CCTA to TEE in excluding the presence of LAA thrombus in patients undergoing PVI in 172 subjects performed during index hospitalization with a median time between studies of <24hours (IQR_25,75_ 0,1 days). statement should read: Mean age was 51 ± 13 years with a median CHADS2 score of 1 (IQR_25,75_ 0,1 range 0–3), and a mean preprocedural INR of 2.1 ± 0.6. Prevalence of an LAA filling defect on single phase CCTA was 9.3% (16/172) versus TEE 1.2% (2/172). The diagnostic accuracy of single phase CCTA is 96.2%. Sensitivity, specificity, positive predictive value, and negative predictive value were 100% (95% CI, 19.8–100%), 91.8% (95% CI, 94–99%), 12.5% (95% CI, 60–76%), and 91.8% (95% CI, 97–100%) for the detection of LAA filling defect, respectively (Table 1).

## 4. Discussion

The study demonstrated that our patient population with atrial fibrillation undergoing PVI may have their LAA excluded with CCTA only. Previous studies on CCTA imaging modalities have already illustrated encouraging results in excluding LAA thrombus. The results of this study demonstrated high sensitivity, specificity, and NPV, that is, 100%, 91.8%, and 91.8%, respectively (Table 2). There were a total of 16 positive filling defects on CCTA of which only two were confirmed with TEE. However, of those 156 single phase studies that did not illustrate any defects, all were validated as negative by TEE (Table 3).

A recent meta-analysis on detection of LAA thrombus and CT in patients with atrial fibrillation reported sensitivity and specificity of 96% and 92%, respectively, with a positive and negative predictive value of 41% and 99% [4]. The high NPV gives the CCTA modality the potential ability to effectively exclude LAA filling defects. As such, a semi-invasive procedure such as a TEE may potentially be avoided. The absence of a filling defect on CCTA strongly negates the need for a subsequent TEE as an adjunct for exclusion of LAA thrombus.

A major limitation to our study was the absence of discriminating LAA thrombus from spontaneous echo contrast (SEC). CT imaging may identify a filling defect that if viewed by TEE will be assessed to be SEC. Shapiro et al. in their retrospective study detail this finding as a false positive. As stated by Shapiro et al., this issue may be addressed with a delayed scan protocol [5]. Furthermore, this issue can further be assessed by using quantitative comparisons of LAA/ascending aorta HU ratios as described by Patel et al. [6]. A study reported a ratio of ≤0.75 as a predictor of LAA thrombus or nonclearing SEC. Other studies having used this ratio to differentiate thrombus from SEC on CCTA, included the categorization of early- and late-phase CT images and comparing thrombus and circulatory stasis and reporting as LAA/AA. Hur et al. evaluated the diagnostic accuracy of a 64-slice CCTA for LAA thrombus in stroke patients using TEE as a standard reference standard [3]. The LAA/Asc Ao (early phase) HU ratios of thrombus and circulatory stasis were 0.12 HU ± 0.12 and 0.19 ± 0.06, respectively, and LAA/Asc Ao (late phase) HU ratios were  0.29 HU ± 0.12 and 0.85 ± 0.12, respectively.

In a prospective study of 500 consecutive AF ablation procedures performed on 424 patients with a mean age of 55 ± 11 years and a CHADS_2_ score mainly between 0 and 1, similar population to ours, the complications rate reported postablation was <1% with zero incidence of death or stroke [7].

## 5. Conclusion

Our findings not only confirms the findings of past studies illustrating the high sensitivity, specificity, and NPV between CCTA and TEE in excluding thrombus, but also confirms that a single phase CCTA if part of planned preparation prior to PVI is sufficient to exclude the presence of thrombus. CCTA should be used as an initial step to exclude the presence of filling defects and complimented by invasive procedures such as TEE for confirmation of an LAA thrombus or delayed phase CCTA in the appropriate clinical setting.

## Figures and Tables

**Table 1:** Baseline demographics of the studied population.

Age	51 ± 13
HTN	89 (51%)
CHF	13 (7.5%)
CVA	6 (3.4%)
DM	24 (13.9%)

**Table 2:** Sensitivity and specificity as well as positive and negative predictive values with their associated confidence intervals.

Sensitivity	100% (95%, CI 28.9–100%)
Specificity	91.8% (95%, CI 87.6–95.9%)
PPV	12.5% (95%, CI 2.5–37.5%)
NPV	91.8% (95%, CI 86.5–95.1%)

**Table 3:** Correlations between CCTA and TEE.

	Positive TEE	Negative TEE	Total
Positive filling defect by CCTA	2	14	16
Positive filling defect by CCTA	0	156	156

Total	2	170	172

## Abbreviations

CCTA: Coronary computed tomography angiography
TTE: Trans thoracic echocardiography
TEE: Transesophageal echocardiography
ASE: American society of echocardiography
PVI: Pulmonary vein isolation
HU: Hounsfield units.
